# INFLUENCE OF TOTAL KNEE REPLACEMENT ON LEAN MASS MEASUREMENTS USING DUAL-ENERGY X-RAY ABSORPTIOMETRY

**DOI:** 10.1093/geroni/igac059.2542

**Published:** 2022-12-20

**Authors:** Jae Young Jang, Miji Kim, Daehyun Lee, Hyung Eun Shin, Chang Won Won

**Affiliations:** Kyung Hee University, Seoul, Seoul-t'ukpyolsi, Republic of Korea; Kyung Hee University, Seoul, Seoul-t'ukpyolsi, Republic of Korea; Kyung Hee Univerisity, Seoul, Seoul-t'ukpyolsi, Republic of Korea; Kyung Hee University, Seoul, Seoul-t'ukpyolsi, Republic of Korea; Kyung Hee University, Seoul, Seoul-t'ukpyolsi, Republic of Korea

## Abstract

Sarcopenia and osteoarthritis often occur together. Many sarcopenic patients with osteoarthritis are managed by total knee replacement (TKR). The prevalence of TKR is increasing in older adults; however, metal implants can lead to the overestimation of lean mass (LM) in dual-energy X-ray absorptiometry (DXA). However, studies considering metal implants in DXA measurement of LM are scarce. Comparisons without and with automatic metal detection (AMD) are important for accurately measuring LM. Therefore, this study examined the effects of TKR on LM. Twenty-four subjects (mean age: 76.4±4.0 years) who underwent TKR were selected from the Korean Frailty and Aging Cohort Study. A GE Lunar iDXA (GE Healthcare Lunar, Madison, WI, USA) system was applied twice (with and without AMD). Leg LM was significantly overestimated in the right and left legs with TKR. The LMs with and without AMD were 6017.1±199.3 g and 5493.7±171.3 g, respectively (p< 0.001), in the right leg, and 5657.1±220.1 g vs. 5173.7±201.8 g, respectively (p< 0.001) in the left leg. The appendicular lean mass index (ALMI) without and with AMD was 6.5±0.6 kg/m2, 6.1±0.6 kg/m2 (p< 0.001). In addition, only one subject was classified as having low muscle mass without AMD, which increased to four based on AMD according to the Asian Working Group of Sarcopenia 2019 guidelines. The overestimated leg LM in subjects after TKR decreased with AMD. Furthermore, LM with AMD increased the prevalence of low appendicular LM in the diagnosis of sarcopenia. Therefore, metal implants should be considered to accurately measure LM using DXA.

